# Screening of known disease genes in congenital scoliosis

**DOI:** 10.1002/mgg3.466

**Published:** 2018-09-09

**Authors:** Kazuki Takeda, Ikuyo Kou, Shuji Mizumoto, Shuhei Yamada, Noriaki Kawakami, Masahiro Nakajima, Nao Otomo, Yoji Ogura, Noriko Miyake, Naomichi Matsumoto, Toshiaki Kotani, Hideki Sudo, Ikuho Yonezawa, Koki Uno, Hiroshi Taneichi, Kei Watanabe, Hideki Shigematsu, Ryo Sugawara, Yuki Taniguchi, Shohei Minami, Masaya Nakamura, Morio Matsumoto, Japan Early Onset Scoliosis Research Group, Kota Watanabe, Shiro Ikegawa

**Affiliations:** ^1^ Laboratory of Bone and Joint Diseases, Center for Integrative Medical Sciences RIKEN Tokyo Japan; ^2^ Department of Orthopaedic Surgery Keio University School of Medicine Tokyo Japan; ^3^ Department of Pathobiochemistry, Faculty of Pharmacy Meijo University Nagoya Japan; ^4^ Department of Women’s and Children’s Health, Dunedin School of Medicine University of Otago Dunedin New Zealand; ^5^ Department of Orthopaedic Surgery Meijo Hospital Nagoya Japan; ^6^ Department of Human Genetics Yokohama City University Graduate School of Medicine Yokohama Japan; ^7^ Department of Orthopaedic Surgery Seirei Sakura Citizen Hospital Sakura Japan; ^8^ Department of Advanced Medicine for Spine and Spinal Cord Disorders Hokkaido University Graduate School of Medicine Sapporo Japan; ^9^ Department of Orthopaedic Surgery Juntendo University School of Medicine Tokyo Japan; ^10^ Department of Orthopaedic Surgery National Hospital Organization, Kobe Medical Center Kobe Japan; ^11^ Department of Orthopaedic Surgery Dokkyo Medical University School of Medicine Mibu Japan; ^12^ Department of Orthopaedic Surgery Niigata University Hospital Niigata Japan; ^13^ Department of Orthopedic Surgery Nara Medical University Kashihara Japan; ^14^ Department of Orthopedics Jichi Medical University Shimotsuke Japan; ^15^ Department of Orthopaedic Surgery, Faculty of Medicine The University of Tokyo Tokyo Japan

**Keywords:** congenital scoliosis, *LFNG*, spondylocostal dysostosis, whole‐exome sequencing

## Abstract

**Background:**

Congenital scoliosis (CS) is defined as a lateral curvature of the spine due to the vertebral malformations and has an incidence of 0.5–1/1,000 births. We previously examined *TBX6* in Japanese CS patients and revealed that approximately 10% of CS was caused by *TBX6* mutations. However, the genetic cause of remaining CS is unknown.

**Methods:**

We recruited 78 CS patients without *TBX6* mutations and major comorbidities, and investigated the genes previously reported to be associated with CS and congenital vertebral malformations by whole‐exome sequencing.

**Results:**

We identified the compound heterozygous missense variants in *LFNG* in one patient. No likely disease‐causing variants were identified in other patients, however. *LFNG* encodes a GlcNAc‐transferase. The *LFNG* variants showed loss of their enzyme function.

**Conclusions:**

A *LFNG* mutation is reported in a case of spondylocostal dysostosis (SCD), a skeletal dysplasia with severe malformations of vertebra and rib. The CS patient with *LFNG* mutations had multiple vertebral malformations including hemivertebrae, butterfly vertebrae, and block vertebrae, and rib malformations. *LFNG* mutations may cause a spectrum of phenotypes including CS and SCD. The current list of known disease genes could explain only a small fraction of genetic cause of CS.

## 1 INTRODUCTION

Congenital scoliosis (CS) is defined as a lateral curvature of the spine due to vertebral malformations; its incidence is 0.5–1/1,000 births worldwide (Giampietro, 2012; Giampietro et al., 2013; Liljenqvist, 2004; McMaster & Ohtsuka, 1982). Segmentation of the vertebrae occurs between 20 and 35 days after conception in human embryonic development (Turnpenny et al., 2007). Somites, the precursors to the ribs, striated muscle, and dermis of the back, are also formed during this period. This process is called somitogenesis. Disruptive perturbation of somitogenesis contributes to congenital vertebral malformations leading to CS. CS is well known to be a heterogeneous disorder ranging from nonsyndromic to possibly syndromic CS who has various major comorbidities (Turnpenny et al., 2007).

Perturbation of somitogenesis causes not only vertebral malformations but also rib malformations. Therefore, many CS patients also have rib malformations. Such a severe form of CS associated with rib malformations is referred to spondylocostal dysostosis (SCD) (Bulman et al., 2000; Giampietro et al., 2006; Sparrow et al., 2006; Sparrow, Guillen‐Navarro, Fatkin, & Dunwoodie, 2008; Whittock et al., 2004). While CS is the view from the orthopedic aspect of the disorder, SCD considers developmental abnormalities of the skeleton (Supporting Information Figure S1). Therefore, some SCD have only mild scoliosis due to the balanced abnormality of the spine even though they have severe vertebral abnormalities. SCD is included in the nosology and classification of genetic skeletal disorder: 2015 revision as the group 35, “Dysostoses with predominant vertebral with and without costal involvement” (Bonafe et al., 2015). Severe spine and rib malformations of CS and SCD usually affect the thoracic growth and function adversely, and lead to thoracic insufficiency syndrome (Campbell et al., 2003; Flynn et al., 2013). Early diagnosis and intervention are mandatory to keep the deformity under control.

Perturbation of somitogenesis resulting in congenital vertebral malformations is etiologically heterogeneous (Giampietro et al., 2013). Several genes associated with congenital vertebral malformations have been identified in human subsequent to identification of model organisms; the examples include *PAX1* (MIM# 167411), *SLC35A3* (MIM# 605632), and *T* (MIM# 601397) (Ghebranious et al., 2008; Giampietro et al., 2005; Thomsen et al., 2006). Whole‐exome sequencing (WES) has identified *DYNC1H1* (MIM# 600112) in a patient with CS and spinal atrophy with lower extremity predominance (Punetha et al., 2015). Notch signaling pathway genes, *DLL3* (MIM# 602768), *MESP2* (MIM# 605195), *LFNG* (MIM# 602576)*, HES7* (MIM# 608059), *RIPPLY2* (MIM# 609891), and *TBX6* (MIM# 602427), are associated with somitogenesis and their mutations have been identified in SCD (Bulman et al., 2000; Giampietro et al., 2006; McInerney‐Leo et al., 2015; Sparrow et al., 2006, 2008, 2013 ; Whittock et al., 2004). It was reported that compound heterozygosity for null mutations and the common hypomorphic risk haplotype composed by three SNPs in *TBX6* caused CS (Wu et al., 2015). Recently, several reports suggested that *TBX6*‐associated CS and SCD may represent a spectrum of a disease caused by the compound heterozygosity model (Lefebvre et al., 2017; Takeda et al., 2017; Wu et al., 2015).

We previously examined *TBX6* in undiagnosed Japanese CS patients and found that approximately 10% of CS was caused by *TBX6* mutations (Takeda et al., 2017). The incidence of *TBX6*‐associated CS was similar worldwide (Lefebvre et al., 2017; Takeda et al., 2017; Wu et al., 2015). However, the genetic cause of CS other than *TBX6* remains unknown. Therefore, we investigated the mutations in genes associated with the development of spine in Japanese CS by WES. We identified the compound heterozygous missense mutations in *LFNG* in one CS patient. These mutations in *LFNG* lead to loss of its enzyme function.

## 2 MATERIALS AND METHODS

### 2.1 Subjects

We recruited 196 Japanese CS patients who were seen at participating hospitals. They received clinical and radiological examinations by expert spinal surgeons and pediatricians. Patients who have known syndromic scoliosis, including Alagille syndrome, Goldenhar's syndrome, Jarcho‐Levin syndrome, Klippel‐Feil syndrome, SOTOS syndrome, and VACTERL association, were excluded. Genomic DNA was extracted from peripheral blood leukocytes using standard methods or from saliva using Oragene DNA collection kit (DNA Genotek, Ottawa) according to the manufacturer's protocol. Informed consent was obtained from the patients and their parents. The study was approved by the ethical committee of all participating hospitals and RIKEN.

### 2.2 WES

*TBX6* mutation was examined as previously described (Takeda et al., 2017). We conducted WES as previously described (Guo et al., 2017; Wang et al., 2017). Briefly, approximately 3 μg DNA was sheared and used for a SureSelect Human All Exon V5 library (Agilent Technologies, Santa Clara, CA) according to the manufacturer's instructions. Patients’ DNA samples were sequenced on a HiSeq2000 (Illumina, San Diego, CA) with 101‐bp paired‐end reads.

### 2.3 Analysis of WES data

Although all patients were sporadic, the mode of inheritance in CS remains still unclear. So, we considered both autosomal dominant and autosomal recessive inheritance. We excluded variants within exons ±30 bp from exon–intron junction that minor allele frequency was above 0.03 in dbSNP137, the National Heart Lung and Blood Institute Exome Sequencing Project Exome Variant Server (NHLBI‐ESP 6500; https://evs.gs.washington.edu/EVS/), Exome Aggregation Consortium (ExAC; https://exac.broadinstitute.org/), Integrative Japanese Genome Variation Database (iJGVD; https://ijgvd.megabank.tohoku.ac.jp/), and our in‐house database from 575 Japanese individuals since the incidence of CS is 0.5–1/1,000 births and SCD is more rare disease than CS worldwide. We conducted the pathogenicity prediction using SIFT (https://sift.bii.a-star.edu.sg/) (Adzhubei et al., 2010), Polyphen‐2 (https://genetics.bwh.harvard.edu/pph2/) (Kumar, Henikoff, & Ng, 2009), and MutationTaster (https://www.mutationtaster.org/) (Schwarz, Cooper, Schuelke, & Seelow, 2014). We defined a variant as “deleterious” when it was predicted as damaging in the three prediction tools. We investigated the variants satisfying the above‐mentioned conditions in previously reported CS and SCD genes: *PAX1*,* SLC35A3*,* T*,* DYNC1H1*,* DLL3*,* LFNG*,* HES7*,* DLL1, MESP2,* and *RIPPLY2*.

### 2.4 DNA sequencing of *LFNG*

The genomic region of the *LFNG* gene including two missense variants (c.467T>G and c.856C>T) was PCR‐amplified using KOD Fx and primers 5′‐CCAGTGCGGCCGCCTGGGTGCTGTGTCAATAAGGGT‐3′ (forward) and 5′‐CCAGTGAATTCATACCGTAGCTCAGCGTCACCTG‐3′ (reverse). The PCR amplicons were cloned into the *Not*I and *EcoR*I sites of the pBluescript SK(‐) cloning vector (Agilent Technologies). After ligation, the vectors and insert DNA fragments were transformed to *E*.*coli* competent cells. The bacterial colonies were collected and verified by Sanger sequencing. *LFNG* variants were detected by direct sequencing of PCR products using KOD Fx (Toyobo, Tokyo) and primer sets using a 3730xl DNA analyzer (Applied Biosystems, Foster City, CA). Nucleotide numbering uses +1 as the A of the ATG translation initiation codon in the reference sequence, with the initiation codon as codon 1.

### 2.5 Expression of the soluble form of the LFNG proteins and enzyme assay of their GlcNAc activity

The expression vector of human LFNG was constructed as described previously with slight modifications (Rampal et al., 2005). A truncated form of LFNG (wild‐type) was amplified from human placenta cDNA (BioChain, Newark, CA) by two‐round PCR. The first PCR was performed with a forward primer, 5′‐GCTGCT CGCCTGCCTGCTGGTGCTCA‐3′, and a reverse primer, 5′‐CTCCACAGAGCA GAACTGCCCAGCAGCCT‐3′, followed by nested PCR with nested primers: a forward primer containing an in‐frame *EcoR*I site located 259‐bp downstream from the initial codon, 5′‐CCGGAATTCAGATGCGGGCCCGCCGCCCGG‐3′, and a reverse primer containing a *Kpn*I site located 3‐bp downstream from the stop codon, 5′‐CGGGGTACCGATTGGGTCTCAGCCATGGC‐3′. Each PCR was conducted with KOD‐Plus DNA polymerase (Toyobo). A DNA fragment, which encoded the human LFNG lacking the first 86 amino acids including the predicted transmembrane domain, was subcloned into a p3xFLAG‐CMV8 (Sigma, St. Louis, MO) vector, resulting in the fusion of LFNG (wild‐type) to the preprotrypsin leader sequence and the 3xFLAG tag sequence at the N‐terminus present in the vector.

Site‐specific mutagenesis of LFNG to produce the p.Leu156Arg and p.Arg286Trp mutants was performed using two rounds of PCR. The first PCR was performed with a 5′‐primer containing an in‐frame *EcoRI* site described above and a 3′‐internal mutagenic oligonucleotide primers (5′‐GCCCGTGTGCCTGGCCCG GGCCTCATCTTCCCC‐3′ or 5′‐GCAGTCATCAGGCAGCCAGATCCGCTC AGCCGT‐3′ for p.Leu156Arg or p.Arg286Trp, respectively), or 5′‐internal mutagenic oligonucleotide primer (5′‐GGGGAAGATGAGGCCCGGGCCAGGCACACG GGC‐3′ or 5′‐ACGGCTGAGCGGATCTGGCTGCCTGATGACTGC‐3′ for p.Leu156Arg or p.Arg286Trp, respectively) and 3′‐primer containing an *EcoR*V site described above, and p3xFLAG‐CMV8/LFNG (wild‐type) as a template. The second PCR was performed with a 5′‐ and 3′‐primers containing a *Hind*III and an *EcoR*V sites described above and KOD‐Plus polymerase, and the first PCR products as a template. The amplified fragments were digested with *EcoR*I and *Kpn*I, inserted into p3xFLAG‐CMV8, and sequenced using a 3730xl DNA Analyzer.

The expression plasmid was transfected into HEK293T cells on a T‐75 flask using FuGENE 6 HD (Promega, Madison, WI). Three days after transfection, 3 ml each of the culture medium and the cells was collected. The cells were lysed with phosphate‐buffered saline containing 0.5% TritonX‐100 and protease inhibitor cocktail (Roche, Basel). Those conditioned media and cell lysates were incubated with 10 µl of anti‐FLAG M2 agarose resin (Sigma) for 2 hr and overnight, respectively, at 4°C. The beads were washed with 1 ml of 50 mM Tris‐HCl, pH 7.5 containing 150 mM NaCl, 0.02% Tween‐20, and then analyzed on a 10% sodium dodecyl sulfate (SDS)‐polyacrylamide gel, transferred to a nitrocellulose membrane (Bio‐Rad), and incubated for 1 hr with anti‐FLAG M2 antibody (Sigma). The bound antibody was detected with anti‐mouse IgG conjugated with a fluorescent dye, IRDye 680RD (Li‐Cor, Lincoln, NA) using ODYSSEY CLx (Li‐Cor). The amount of recombinant LFNG protein was estimated by standard curve from fluorescent intensity of 3xFLAG‐tagged bovine alkaline phosphatase (Sigma) using Image Studio (ver 4.0, Li‐Cor).

GlcNAc‐transferase activity was examined as described previously (Rampal et al., 2005). Briefly, the GlcNAc‐transferase assay mixture contained 10 µl of enzyme‐bound anti‐FLAG affinity resins, 50 mM 2‐(N‐morpholino) ethanesulfonic acid‐NaOH (pH 6.5), 10 mM MnCl_2_, 0.1 mM UDP‐GlcNAc (ultrapure grade, Promega) as the sugar donor substrate, and 1 mM *p*‐nitrophenyl‐α‐l‐fucose (pNP‐Fuc, Sigma) as the sugar acceptor in a total volume of 50 µl. The reaction mixture was incubated at 37°C for 2–4 hr. The reaction product, UDP moiety released from UDP‐GlcNAc, was mixed with UDP detection reagent, which contains an enzyme converting UDP to ATP, in UDP‐Glo™ Glycosyltransferase Assay kit (Promega). The newly synthesized ATP to be measured using a luciferase/luciferin reaction and the luminescent signals were detected using a luminometer, Victor X4 (PerkinElmer, Waltham, MA).

## 3 RESULTS

### 3.1 Clinical features of the CS patients

We found *TBX6* mutations in 15 out of 196 CS patients. From the patients without *TBX6* mutations, we selected 78 nonsyndromic CS patients (Supporting Information Table S1) and examined them by using WES. Their mean age was 12.0 ± 5.4 years old. Ten patients had family histories of scoliosis; two had CS in their families. The mean Cobb angle of the main curve was 43.0º ± 13.4º. Eight patients had various comorbidities. Various types of rib malformations were found in 23 patients; one irregular shaped, 11 fused, six missing, and five additional ribs.

### 3.2 WES

**Figure 1:**
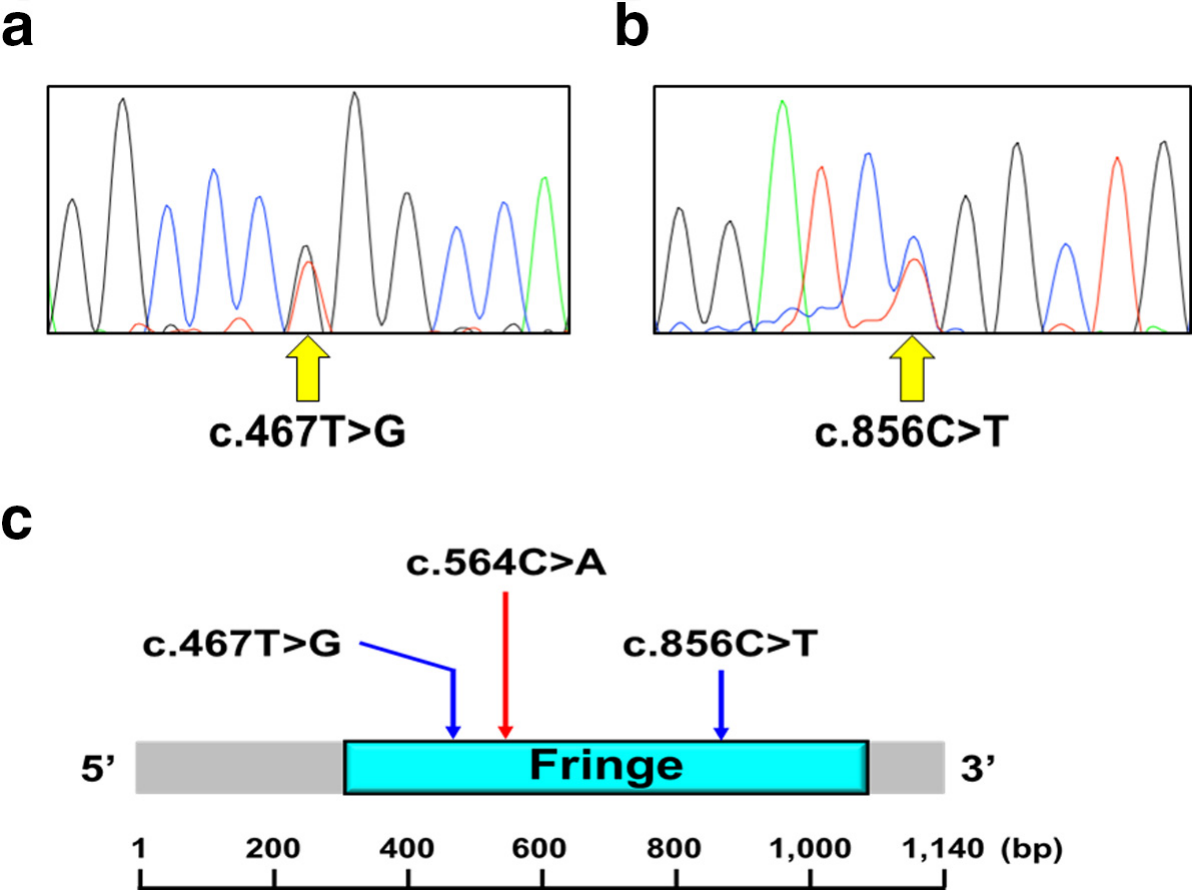
*LFNG* mutations in a patient with congenital scoliosis. (a,b) Direct sequencing of PCR amplicons from the patient's genomic DNA showing: (a) c.467T>G (p.Leu156Arg) and (b) c.856C>T (p.Arg286Trp). Nucleotide numbering uses +1 as the A of the ATG translation initiation codon in the reference sequence, with the initiation codon as codon 1. (c) The positions of the missense variants in the *LFNG* cDNA. c.467T>G and c.856C>T were located in the Fringe domain. Previously reported missense mutation, c.564C>A, was also located in the domain

The mean depth of coverage for reads was 74.3×, and 94.3% of the targeted bases had more than 20 reads on average (Supporting Information Table S2). By evaluation of the variants for their pathogenicity, we identified a CS patient (S1289) who had two likely disease‐causing variants in *LFNG*: c.467T>G (p.Leu156Arg) and c.856C>T (p.Arg286Trp) (Table 1). The sequences of the variants were confirmed by Sanger sequencing (Figure 1a,b). We did not identify any likely disease‐causing variants in other genes (*PAX1*,* SLC35A3*,* T*,* DYNC1H1*,* DLL3*,* HES7*,* DLL1*,* MESP2*, and *RIPPLY2)*. The coverages of the exons of these genes which had more than 10 reads on average were over 90% (Supporting Information Table S3).

**Table 1:** In silico evaluation of pathogenicity of the missense variants identified in S1289

Gene	Alteration		Allele frequency				Mutation prediction		
	Nucleotide	Amino Acid	dbSNP	esp6500	ExAC	iJGVD	SIFT	PolyPhen‐2	MutationTaster
*LFNG*	c.467T>G	p.Leu156Arg	–	–	–	3.00E‐04	0	Probably D	DC
	c.856C>T	p.Arg286Trp	rs752671299	–	2.50E‐05	–	0	Probably D	DC

Note

DC: disease causing: ExAC: Exome Aggregation Consortium; iJGVD: Integrative Japanese Genome Variation Database; Probably D: probably damaging.

### 3.3 Evaluation of *LFNG* variants

c.467T>G and c.856C>T in *LFNG* had been annotated in ExAC and iJGVD; allele frequency of c.467T>G was 3.0 × 10^−4^ in iJGVD and that of c.856C>T was 2.5 × 10^−5^ in ExAC. Both variants were predicted to be disease causing by SIFT, Polyphen‐2, and MutationTaster (Table 1). Previously, only one *LFNG* mutation (c.564C>A; p.Phe188Leu) has been reported in a SCD patient.

Because the parents’ DNAs were unavailable, we examined the haplotype of the two missense variants in S1289 by a long‐range PCR followed by sequencing the subcloned amplicons. We found that S1289 was compound heterozygotes for the two missense variants.

### 3.4 Functional analysis of *LFNG* variants in vitro

**Figure 2:**
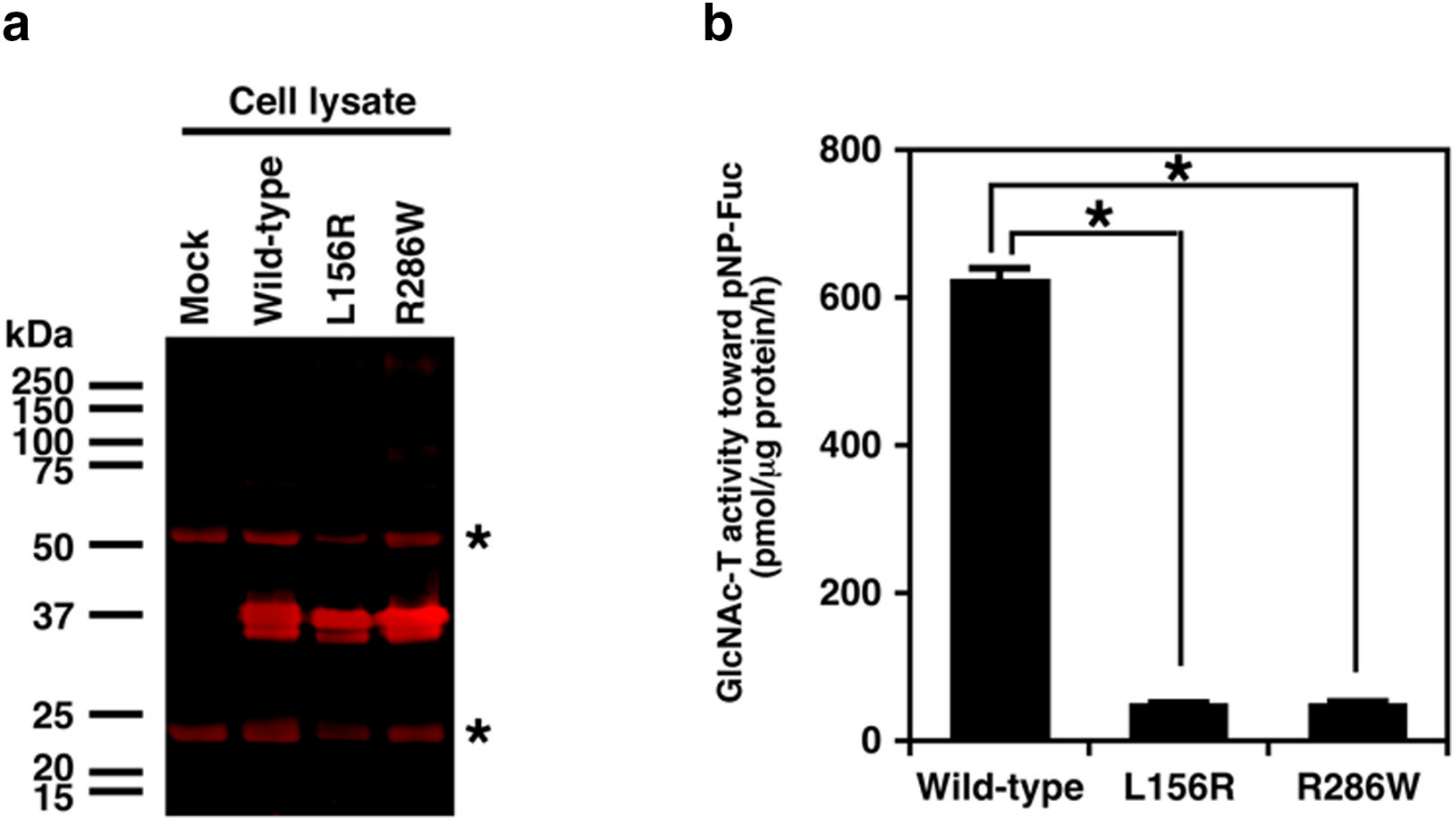
Protein level and GlcNAc‐transferase activity of recombinant LFNG expressed in HEK293T cells. (a) Western blot analysis of the recombinant LFNG proteins: wild‐type, L156R (p.Leu156Arg), and R286W (p.Arg286Trp). The purified recombinant LFNG from cell lysate was detected with the anti‐FLAG and fluorescence‐conjugated anti‐mouse IgG antibodies. Asterisks indicate heavy and light chains of the anti‐FLAG antibody from the anti‐FLAG agarose resin for purification. (b) GlcNAc‐transferase activity of the recombinant LFNG in the cell lysate. Both L156R and R286W showed significantly decreased enzyme activities. Values are the means ± *SE* (*n* = 3). *p < 0.0001 versus the wild‐type was calculated by the Student's *t* test

To examine whether the *LFNG* missense variants, p.Leu156Arg and p.Arg286Trp cause a reduction of the GlcNAc‐transferase activity, the LFNG proteins in soluble forms with a 3xFLAG epitope were generated by replacing the first 86 amino acids of LFNG with a cleavable pre‐pro‐trypsin signal sequence. The soluble 3xFLAG‐tagged LFNG proteins were expressed in HEK293T cells. The recombinant enzymes secreted in the medium were adsorbed onto anti‐FLAG antibody‐bound beads to eliminate endogenous LFNG and to facilitate the purification of the enzyme. The expression levels of wild‐type and a mutant enzyme (p.Arg286Trp‐ and p.Leu156Arg‐LFNG) in the cell fraction were comparable between the wild‐type and mutants (Figure 2a). GlcNAc‐transferase activities of both mutants from cell lysate were significantly reduced compared to that of the wild‐type LFNG (Figure 2b). These data indicate that the two LFNG variants lead to loss of the enzyme function. By contrast, the expression of wild‐type and a mutant enzyme, p.Arg286Trp‐LFNG, but not p.Leu156Arg‐LFNG, was detected in the conditioned medium (Supporting Information Figure S2a). These observations suggest that the secretion pathway of p.Leu156Arg‐LFNG might be impaired. The GlcNAc‐transferase activity of p.Arg286Trp‐LFNG from the conditioned medium was also drastically decreased compared to that of the wild‐type LFNG (Supporting Information Figure S2b).

### 3.5 Clinical features of the patient with *LFNG* mutations

**Figure 3:**
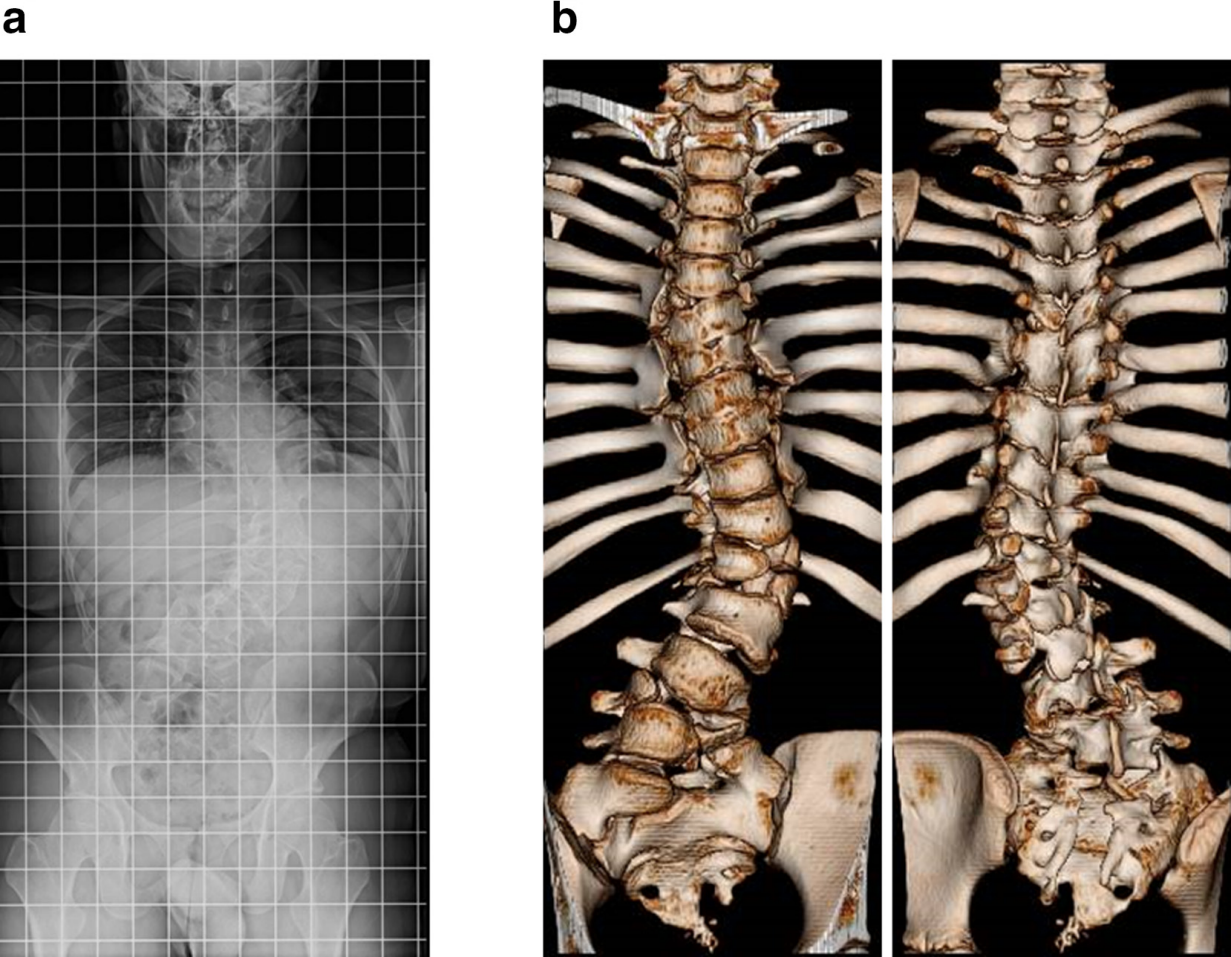
Radiographic phenotype of the congenital scoliosis patient with *LFNG* mutations. (a) The standing spinal antero‐posterior radiograph. The patient has severe scoliosis with a Cobb angle of 70° at T9‐L3. (b) Three‐dimensional CT (left: front view, right: back view). Multiple congenital vertebral malformations including hemivertebrae, block vertebrae, and butterfly vertebrae are noted. Multiple rib malformations including hypoplasia and fused ribs are also found

S1289 was a 16 years old male who was born as a child of nonconsanguineous healthy parents. He presented with mild short stature (−2.1 *SD*) and had no comorbidity (Supporting Information Table S1). He had severe scoliosis and was diagnosed with CS. The main Cobb angle was 70° at T9‐L3 (Figure 3a). He had multiple vertebral malformations: hemivertebrae at T5, L1, and L4 (one segmented type and two nonsegmented type), block vertebra at T8‐T9, and butterfly vertebrae (nonsegmented type) at T7, T12, and L5 (Figure 3b). Multiple rib malformations, including hypoplasia of bilateral 2nd ribs and left 6–7th, 8–9th, right 5–6th, 7–8th, and 9–10th fused ribs, were also found.

## 4 DISCUSSION

Our WES identified a CS patient who is compound heterozygous for two novel missense variants of *LFNG*. Only one *LFNG* mutation (c.564C>A, p.Phe188Leu) has previously been reported, which causes SCD3 (MIM# 609813) in the homozygous state (Sparrow et al., 2006). Both of the variants we found were rare and were predicted deleterious in all bioinformatic tools examined. *LFNG* encodes an O‐fucosylpeptide 3‐β‐*N*‐acetylglucosaminyltransferase that adds *N*‐acetylglucosamine (GlcNAc) residues to O‐fucose on the EGF‐like repeats of Notch receptors (Bruckner, Perez, Clausen, & Cohen, 2000; Moloney et al., 2000). Our variants and the previously reported mutation are all located in highly conserved region that encodes a glucosaminyl‐transferase; particularly, the Arg286 residue where we found the mutation was conserved in all known fringe proteins from *Drosophila melanogaster* to human (Correia et al., 2003). Although these in silico evaluation data strongly suggested that the two variants are disease‐causing mutations, it has been reported that to predict the deleterious effect of missense variants in silico is sometimes difficult (Majithia et al., 2016; Takeda et al., 2017). Therefore, we examined the effect of the variants in vitro and revealed that both lead to loss of the GlcNAc‐transferase activity. Based on these observations, the two *LFNG* variants are loss of function mutations and hence are pathogenic to the CS phenotype. p.Leu156Arg was suggested to cause mislocalization of the mutant protein in addition to its loss of enzymic activity (Supporting Information Figure S2a). It is reported that p.Phe188Leu mutation was also mislocalized and enzymatically inactive by a functional assay (Sparrow et al., 2006).

The CS patient who carried the *LFNG* mutations (c.467T>G; p.Leu156Arg and c.856C>T; p.Arg286Trp) had severe scoliosis due to interspersed vertebral malformations from thoracic to lumbar spine together with multiple rib malformations, while the SCD patient who carried the *LFNG* mutation (c.564C>A; p.Phe188Leu) had nonprogressive scoliosis but more extensive and a large number of congenital vertebral malformations like “pebble beach” from cervical to lumbar spine as well as severe short trunk and finger abnormalities (Sparrow et al., 2006; Turnpenny et al., 2003). Thus, *LFNG* mutations are considered to cause a spectrum of vertebral malformation recognized as CS and/or SCD (Supporting Information Figure S1). Such phenotypic variation including CS and SCD is also known in vertebral malformation caused by *TBX6* mutations (Lefebvre et al., 2017; Takeda et al., 2017). Further, accumulation of the patients with *LFNG* mutations is mandatory to conclude the phenotypic range of the *LFNG* mutation and genotype–phenotype association.

In the screenings of SCD genes, known gene mutations have been found in 20%–25% of the patients examined; 60% of SCD is caused by *DLL3* mutations (Bonafe, Giunta, Gassner, Steinmann, & Superti‐Furga, 2003; Turnpenny et al., 2003; Turnpenny, Sloman, & Dunwoodie, 1993; Whittock et al., 2004). In contrast, our screening of known disease genes for CS and vertebral malformation could only find the causal genes in <10% of nonsyndromic CS patients who had no major comorbidities; CS in our cohort did not have the mutations in known genes causing CS and SCD other than *TBX6* and *LNFG*. CS is a very heterogeneous disorder; the type, location, number of vertebral malformations, and comorbidities are different between the patients (Giampietro et al., 2013). On the other hand, CS caused by the *TBX6* mutation has been reported to have specific abnormality of the vertebra (Takeda et al., 2017; Wu et al., 2015). The vertebral anomaly along the whole length of the spine with the vertebrae of rounded shape (“pebble beach” sign) is a feature of SCD1 (MIM# 277300), which is caused by *DLL3* mutations (Turnpenny et al., 2003). Their clinical differences suggest considerable genetic heterogeneity of CS. In the current study, we investigated the limited number of genes that are considered to be associated with the development of spine. Most of them are related to the Notch signal. Somitogenesis of vertebrate species relies on the intersecting gradients and cross‐regulatory activities of various signal transduction pathways, not only Notch signaling but also Fgf, Wnt, and retinoic acid signaling pathways (Gibb, Maroto, & Dale, 2010; Wahi, Bochter, & Cole, 2016). It was reported that mutations in *Wnt3a* identified as a negative regulator on Notch signaling and somitogenesis associated with congenital vertebral malformations (Aulehla et al., 2003; Hayward, Kalmar, & Arias, 2008). Therefore, further comprehensive approach might identify the causal genes for CS. However, our preliminary screening of unknown CS gene(s) for the exome data did not present a possible causal gene that had likely deleterious mutation in more than two cases. To discover the unknown causal genes for the rare diseases by WES is still challenging and tends to come up against the *N* = 1 problem (Akawi et al., 2015). It would be mandatory to increase the sample size and evaluate the phenotype well.

## CONFLICT OF INTEREST

The authors declare no conflict of interest associated with this manuscript.
