# Supplementary material for: Screening of known disease genes in congenital scoliosis
Source: Mol Genet Genomic Med. 2018 Sep 9;6(6):966–74. doi: 10.1002/mgg3.466 (PMC6305645; doi:10.1002/mgg3.466)
Supplement: Supplementary file 1 [file MGG3-6-966-s001.docx]

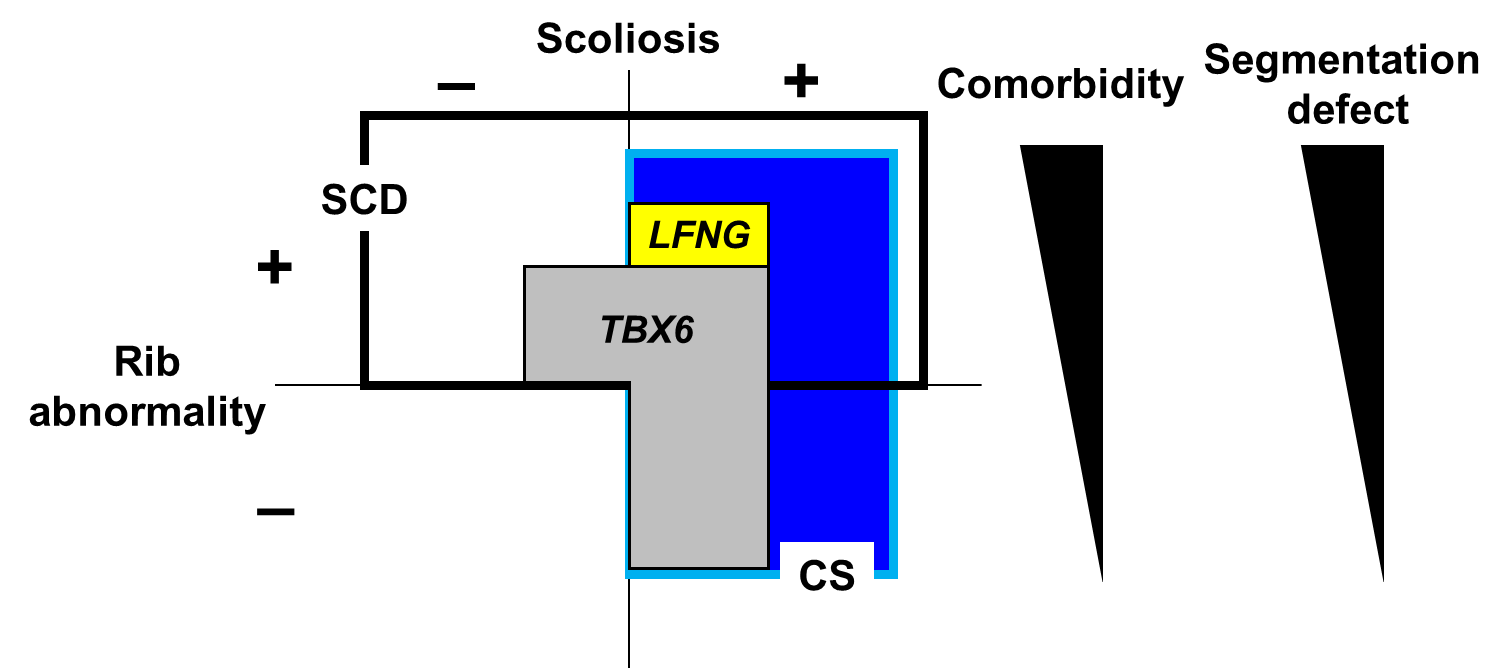


**Figure S1. The disease concept of Congenital scoliosis and Spondylocostal dysostosis.**

CS: Congenital scoliosis, SCD: Spondylocostal dysostosis, TBX6: patients with *TBX6* mutations*,* LNFG: patients with *LNFG* mutations. CS and SCD are overlapping, but distinct disease concepts. Both are vertebral segmentation disorders. CS refers to the scoliosis caused by vertebral mal-segmentation; SCD refers to skeletal dysplasia affecting vertebrae and ribs. Rib abnormality is absent in many cases of CS while it is an essential component of SCD. Many SCD patients do not present scoliosis because of 'balanced' vertebral abnormality. Mutations of *TBX6* and *LNFG* produce a spectrum of phenotype including CS and SCD. In SCD, mutations of *DLL3*, *HES7* and *MESP2* are also found.


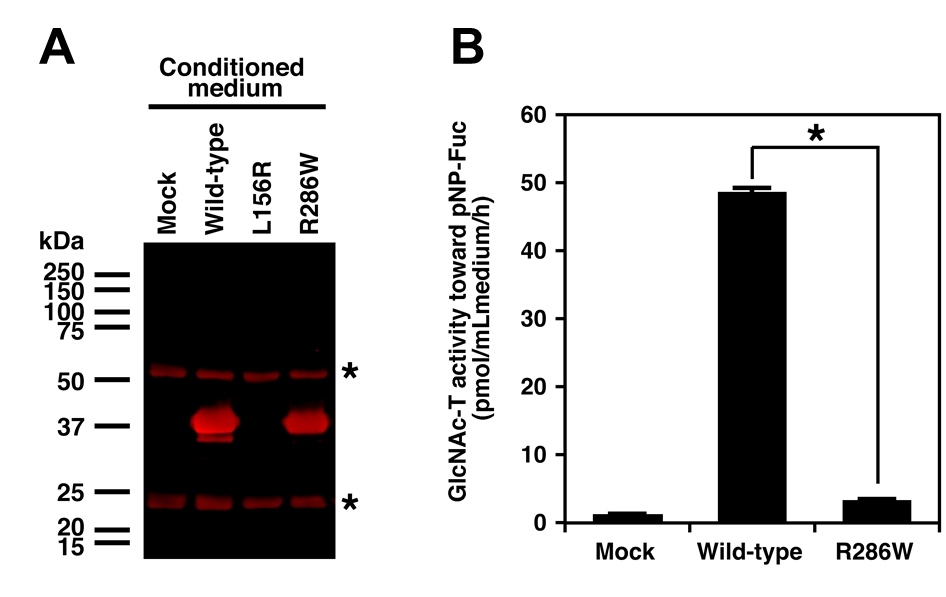


**Figure S2. Protein level and GlcNAc-transferase activity of recombinant LFNG expressed in HEK293T cells.**

(A) Western blot analysis of the recombinant LFNG (wild-type, L156R and R286W). The purified recombinant LFNG from conditioned medium separated by SDS-PAGE was detected with the anti-FLAG and a fluorescence-conjugated anti-mouse IgG antibodies. The broad signals of the FLAG-tagged LFNG (~36 kDa) may be due to N-glycosylation of enzymes. Protein levels of recombinant LFNGs were estimated by the fluorescence intensity compared to standard curve generated from concentrated known 3xFLAG-bovine alkaline phosphatase. Asterisks indicate the heavy and light chains of anti-FLAG antibody from the anti-FLAG agarose resin for purification. L156R- LFNG was not secreted in the conditioned medium.

(B) GlcNAc-transferase activity of the recombinant LFNG from conditioned medium. GlcNAc-transferase activity of each enzyme protein was examined using the partially purified recombinant enzymes from conditioned medium, UDP-GlcNAc as the sugar donor substrate, and pNP-Fuc as the sugar acceptor substrate. GlcNAc-transferase activity was determined by measurement of level of UDP released from UDP-GlcNAc. “Mock” indicates the result by the enzyme source from the HEK293T cells without transfecttion. Values are the means ± SE (n=3). *P < 0.0001 versus wild-type was calculated by the Student’s t-test.
